# Deep Neural Network for EEG Signal-Based Subject-Independent Imaginary Mental Task Classification

**DOI:** 10.3390/diagnostics13040640

**Published:** 2023-02-09

**Authors:** Farheen Siddiqui, Awwab Mohammad, M. Afshar Alam, Sameena Naaz, Parul Agarwal, Shahab Saquib Sohail, Dag Øivind Madsen

**Affiliations:** 1 Department of Computer Science and Engineering, School of Engineering Sciences and Technology, Jamia Hamdard, New Delhi 110062, India; 2 Department of Business, Marketing and Law, USN School of Business, University of South-Eastern Norway, 3511 Hønefoss, Norway

**Keywords:** electroencephalography, deep neural network, principal component analysis, mental task, feature extraction

## Abstract

BACKGROUND. Mental task identification using electroencephalography (EEG) signals is required for patients with limited or no motor movements. A subject-independent mental task classification framework can be applied to identify the mental task of a subject with no available training statistics. Deep learning frameworks are popular among researchers for analyzing both spatial and time series data, making them well-suited for classifying EEG signals. METHOD. In this paper, a deep neural network model is proposed for mental task classification for an imagined task from EEG signal data. Pre-computed features of EEG signals were obtained after raw EEG signals acquired from the subjects were spatially filtered by applying the Laplacian surface. To handle high-dimensional data, principal component analysis (PCA) was performed which helps in the extraction of most discriminating features from input vectors. RESULT. The proposed model is non-invasive and aims to extract mental task-specific features from EEG data acquired from a particular subject. The training was performed on the average combined Power Spectrum Density (PSD) values of all but one subject. The performance of the proposed model based on a deep neural network (DNN) was evaluated using a benchmark dataset. We achieved 77.62% accuracy. CONCLUSION. The performance and comparison analysis with the related existing works validated that the proposed cross-subject classification framework outperforms the state-of-the-art algorithm in terms of performing an accurate mental task from EEG signals.

## 1. Introduction

EEG classification signals have been widely used in different cognitive science and healthcare applications. This includes brain computer interface (BCI) studies, neuroscience and neurocognitive applications, mental task classification, etc. An effective application of EEG is to classify mental tasks while subjects are known and available, i.e., subject-dependent mental task classification. Moreover, researchers are looking at subject-independent mental task classifications. EEG plays a vital role in establishing interaction between various areas and hence analyses of the consequences of diseases on brain functioning suggest BCI for paraplegic individuals [1,2]. The BCI is based on recorded EEG signals from brain activity together with computational inferences. With upcoming accurate EEG data collection techniques, researchers have developed new frameworks to analyze the changes in the brain functioning of patients [3] at the time of the treatment. Therefore, future research of BCIs for people with health alignments is based on EEG signals that help them utilize existing mental and motor capabilities to regulate the system [4,5]. With this, the patient would be able to operate and eventually control support systems such as artificial limbs and wheelchairs.

With cross-subject EEG training of these devices, the patient EEG data used by the devices will not be mandatory for the training phase. Several researchers have applied various classification methods for mental task classification. Manali et al. [6] have proposed a mental task classification using variational mode decomposition (VMD) to extract features from the single-channel EEG. There were three stages of processing in their work. They first decomposed the signal using VMD and then calculated the variational mode energy ratio proposed in their work followed by an adaptive boosting algorithm for the classification purpose. Feature reduction is a crucial step in any machine learning task and has been studied by Conrado et al. [7] for the classification of mental tasks using ANNs. The convolutional neural network (CNN) has also been widely used by many researchers. In their work, Pallavi et al. [8] studied the image processing capability of a CNN. They used the scalogram images of EEG data for the classification of different emotions. The model developed by these authors was tested for different datasets and was found to be subject-independent.

The Bidirectional Long Short-Term Memory Network (BiLSTM) proposed by Jinru et al. [9] was also used for the classification of various emotions using EEG signals. EEGNet [10] is another CNN-based model developed for EEG-based BCIs. In their work, the authors used depth-wise and separable convolutions for model development. They compared the results obtained for cross-subject and within-subject classifications with the different approaches across the four BCI paradigms, namely, P300 visual-evoked potentials, ERN, MRCP, and SMR. Madhuri et al. [11] classified hand movement and word generation using a Hierarchical classifier that employed optimized Neural Networks on the EEG signals.

Deep Learning Network has been used to study the correlation between various features of input signals by Suwicha et al. [12]. In 2014, Xiu et al. [13] applied the DL algorithm for the classification of EEG data extracted for the Motor Imagery task (MIT). The two tasks studied by these researchers were the imagination of the left hand and the right hand motor activities. Saadat et al. used Back Propagation Neural Network (BPNN) along with the Hidden Markov Model (HMM) [14] for the classification of mental tasks. The design of brain interfaces used by patients with neural disorders to communicate and control various devices has been studied by Hema et al. [15,16]. They have proposed a particle swarm optimization (PSO) algorithm for training the functional link neural network for the classification of the EEG signals obtained from two subjects for five different mental tasks. In their work, Jose et al. [17] studied various online learning mechanisms used in brain–computer interfaces (BCI) that can help in obtaining fixed learning rates in patients with neural disorders.

Debarshi et al. [18] studied subject-independent and subject-dependent models separately for EEG-based emotion detection and classification. From this work, they concluded that conventional machine learning techniques work better in the case of subject-independent decision making. The features have been extracted from the Power Spectral Density (PSD) of the obtained EEG data and were combined with the Support Vector Machine by the authors in [19] for the classification of subjects as happy and unhappy. Linear Discriminant Analysis (LDA) together with Common Spatial Patterns (CSP) achieved extraction of relevant features and classification by the authors in [20]. An ensemble classifier is formed by combining multiple classifiers with 11 different regression expressions. However, since the hand-crafted features being used in these methods have very little ability, the learning strategies being employed are also traditional. Hence, the performance is quite poor. These cross-subject problems with large and complex data can be handled in a much better way by employing deep learning techniques [10,21]. The studies reported in [22,23,24,25,26] quantified EEG features to recognize neurological deteriorations according to the task because of stroke and estimate the biomarkers to differentiate between healthy adults and ischemic stroke patients.

The applications of EEG-based mental task classification have grown considerably recently. However, subject-dependent mental classification is widely used and subject-independent mental task classification has yet to be well explored by researchers. To this end, the main contribution and novelty of the current article is the classification of mental tasks by averaging subjects’ task using the power spectral density (PSD). Since the EEG signals are very random and have high variance, averaging aided in obtaining better accuracy. In addition to this, we have achieved an accuracy which outperforms state-of-the-art approaches.

The rest of the paper is arranged as follows: Section 2 focuses on the core concepts employed in this research. The proposed model is discussed in Section 3 with a clear block diagram illustrating each step clearly, and the experimental results are discussed in Section 4. Section 5 explicitly discusses these results and conclusions and limitations of the work are covered in Section 6.

## 2. Background

### 2.1. Feature Extraction

Feature extraction can be performed by using the power spectral density (PSD) of any signal. This method is specifically suited for narrow band signals. By using this technique, the signal power is distributed over a range of frequencies and helps us in obtaining an estimate of spectral density from the dataset. To obtain the PSD, the autocorrelation function of the signal was calculated, followed by calculating its Fourier transform (FT). Here, the signal was perceived as a random sequence that was used to determine its power. The unit of measurement for power spectral density is watts per hertz (W/Hz). PSD is a frequency domain analysis in which a signal is decomposed into smaller sub-signals and it can be categorized as parametric, non-parametric, and subspace. In the parametric approach, the system parameters are calculated under the assumption that the presence of white noise influences the output of any linear system. Burg’s method [27] and Yule–Walker’s AR [28] method are examples of this approach. Non-parametric approaches are computationally less expensive and robust but as they cannot extrapolate the finite length sequence beyond the signal length so the frequency resolution is not very good. They also suffer from the drawback of spectral leakage [29]. Some non-parametric approaches are the Bartlett window, Periodogram-based estimation, and Welch window. The subspace method is the preferred choice for signals that have a low signal-to-noise ratio (SNR). In this method, the PSD is obtained by calculating the Eigen decomposition of the autocorrelation matrix. This is a preferred choice for linear and sinusoidal signals, but it does not give the true PSD values.

The EEG signal data are highly dimensional and hence dimensionality reduction methods are needed before using them for any machine learning model. PCA (Principal Component Analysis) is a well-established method in the literature for extraction of relevant features and hence reducing dimensions of the dataset. During PCA, the original signal data in matrix form are used to calculate covariance in the dataset [30]. Many linear transformations are applied for this purpose [31] and finally, eigenvectors and eigenvalues are obtained [32]. The largest eigenvalue obtained corresponds to the most discriminating feature. Therefore, features having discriminating power are retained, and unimportant features can be ignored. PCA is the most widely used technique to reduce the dimensions of EEG data [33].

### 2.2. Deep Neural Network

A Deep Neural Network is designed with multi-hidden layers as opposed to a single neuron network containing a single hidden layer. This means that the input data undergo a non-linear transformation at multiple layers to produce the output. It uses algorithms such as Stochastic Gradient Descent (SGD) and its variations for error estimation for the current model state. Based on error estimation, the weights of the models are updated. Artificial Neural Networks comprise weights between the neurons at the hidden layer and the input and output layers which have to be fine-tuned to improve the model. Gradient descent was used here during backpropagation to minimize the error. Stochastic Gradient Descent is a more simplified and efficient version of Gradient Descent as it takes only a random subsample of the total available data to calculate the error function. As only a subset of the complete dataset was used here, it can be used to train a very large dataset even if there are memory constraints. Another advantage is that it is convex in nature and avoids local minima and plateaus. In their work, [34] added a momentum term that showed better performance in terms of convergence speed in the training of deep neural networks. The value of the latest calculated gradient term influenced the next gradient value calculation due to the addition of this momentum.

Adaptive Moment Estimation (ADAM) is another algorithm derived from SDG that has an adaptive learning rate for each parameter [35]. This algorithm trains the model more efficiently, but also requires more memory. Another important part of ANNs is the activation function being used. Traditionally, Sigmoid activation has been the most commonly used function. When this function is used for training a Deep Neural Network, it suffers from a problem known as the vanishing gradient problem. Due to this problem, the DNN or RNN is not able to backpropagate the gradient value toward the layers closer to the input layer. This results in the poor learning ability of a model, and hence premature convergence. A new activation function, Rectified Linear Unit (ReLU), has been incorporated which gives the output as 0 if the input is below 0 and outputs the input itself if it is above 0. This function is now most commonly used in DNNs as it solves the problem of the vanishing gradient in a very efficient manner [36]. Traditionally, artificial neural networks are fully connected in nature. These fully connected layers require a huge amount of computation as the number of inputs increases and hence it has poor scalability. Apart from these dense or fully connected layers, deep neural networks have many other types of layers such as a convolutional layer, pooling layer, recurrent layer, etc. Each of these layers performs differently and hence is best suited for different types of applications.

### 2.3. EEG Data Acquisition

**Figure 1:**
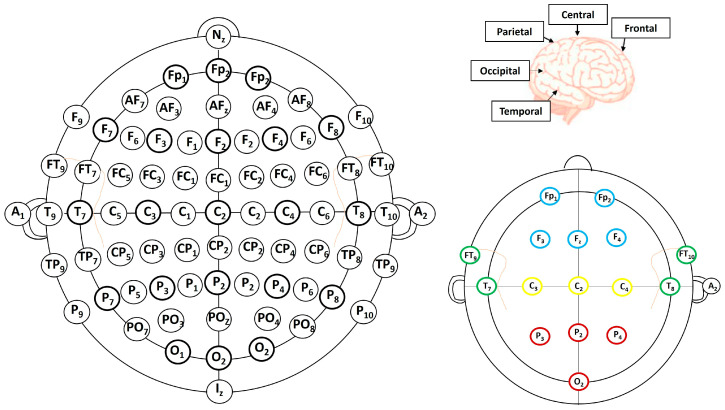
Brain lobes and electrode placements [34].

Electroencephalography is a non-invasive procedure that represents captured electrical potential by attaching electrodes to a subject’s scalp [37]. This instantaneous propagation of voltage changes results in high-precision temporal information acquisition by the EEG. That is why most researchers are using EEG data. Limited spatial resolution is achieved through EEG as the human skull and scalp act as insulators affecting the dispersal of the signal. However, the EEG signal acquisition process is not very expensive and does not require protection since the magnetically shielded closed output of EEG is one time series corresponding with a channel (between 32 and 256). Each of the time series represents the electrical potential on the subject’s scalp. These channels are placed concerning a reference electrode and signals are recorded at rates from 250 Hz to 1000 Hz. Five categories of EEG frequency bands are generally referred to: frequencies less than 4 Hz are placed in the delta band, the frequency range of 4–8 Hz is the theta band, the alpha band is the frequency range of 8–14 Hz, the beta band is the frequency range of 14–40 Hz and frequencies above 40 Hz are placed in the gamma band. The recording of EEG signals can be done in mono-polar mode or bipolar mode. The monopolar recording is carried out by observing the voltage difference between the reference electrode and the scalp position where an electrode is placed. The position of the reference electrode is fixed, usually near the human ear lobe. On the contrary, during the bipolar mode of recording, the difference in the electrode voltages of two scalp electrodes is observed. For recording EEGs, the subject wears an electrode cap having electrodes placed as specified by the “10/20 international electrode placement system” [38] depicted in Figure 1.

The international system establishes the constraint that contiguous electrodes must be at a distance of either ten percent or 20% of the skull. This distance is the total distance from the front to the back or the distance from the left to the right of the skull. The area of the head is divided into various lobes. Letters are used to represent various positions of the lobes. The cerebral cortex appears to be the outermost layer of the brain. Vertically splitting the brain shows two cerebral hemispheres (lengthwise). Each of these hemispheres is divided further into four lobes: frontal, parietal, temporal, and occipital. The frontal lobe is in charge of a variety of tasks including body mood regulation, problem-solving, and planning. The parietal lobe is responsible for the integration of sensory information. Sensory information such as hearing memory and language recognition is processed by the temporal lobe. The occipital lobe of the brain is where most visual processing takes place.

### 2.4. Dataset

The dataset used in this work was taken from BCI competition III dataset V. The authors in [17] generated the dataset by recording the EEG potential at electrode positions according to the International 10–20 system using a Biosemi system and a cap. The EEG signals in BCIs have been shown in various works [39,40,41]. The mental tasks performed in [17] were:

i. Subject imagining self-paced movements performed with the left hand repetitively;
ii. Subject imagining self-paced movements performed with the right hand repetitively;
iii. Subject performing word generation of words starting with the same letter.

**Figure 2:**
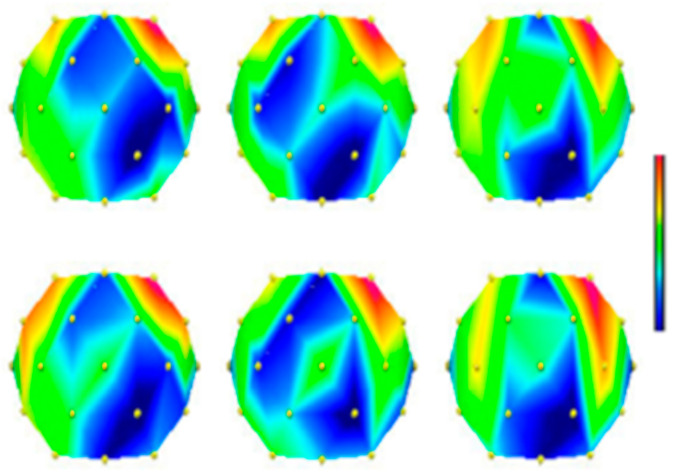
Power maps of subject 2 for two consecutive recordings [17] for all three considered tasks. In addition, the allotted band was 8–12 hertz, where electrode’s positions are represented by filled circles.

Figure 2 shows the brain power maps captured in the frequency range between 8 and 12 Hz. These maps correspond to the three above-stated imagined mental tasks of the BCI competition III dataset V belonging to one subject. These maps were taken by [17] for two consecutive recording sessions that are shown in the top panels and bottom panels of Figure 2, respectively. The mean value of all the EEG data for a particular mental task was calculated as movements (left panels) and then used to make maps. The left panel depicts the imagination of the left hand. The imagination of the right hand movements is shown in the central panels while the task of imagining word generation starting from the same random letter corresponds to the right panels in Figure 2. In this figure, the filled circles represent the electrode placement (frontal on top). Figure 2 shows that the brain maps of the given imaginary mental tasks and the corresponding EEG data showed similar results in different sessions. Specifically, this can be observed for the task of imagining left hand and right hand movements. It was observed in [17] that the power map of the “right” task recorded during the second session was similar to the power map of the “left” task recorded during the first session. This shows that the variability present in the EEG data between different sessions hampers accurate predictions. 

## 3. Proposed Methodology

**Figure 3:**
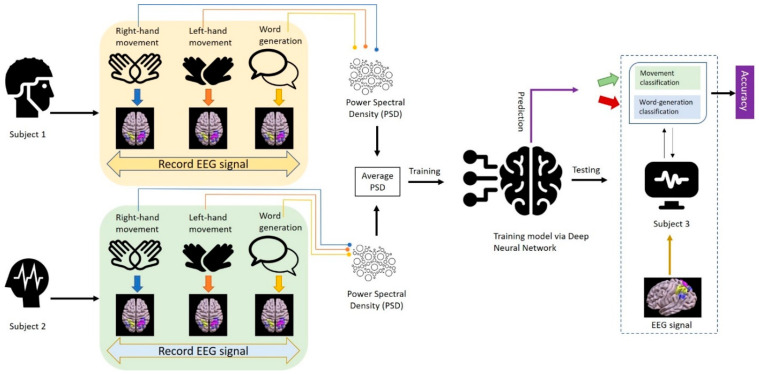
Flowchart for the proposed model.

In this section, the proposed methodology is described stepwise. First, a description of the data is given, and then the data pre-processing is explained, followed by the architecture of the methodology (model) in Figure 3.

### 3.1. Dataset Description

The dataset for imagined mental tasks from the BCI competition III dataset V was used in this work. There are three training files for the first three sessions and one testing file (corresponding to the fourth recording session). The training files are labelled and hence were used in the training phase of supervised learning while testing files in the dataset were without labels. Data are provided in ASCII format. The dataset provides raw EEG signals as well as pre-computed features of the EEG data. In this research, the pre-computed features data file was used for experimentation. The pre-computed feature files contain a PSD sample per row. The number of PSD samples for the three subjects is given in Table 1. The 97th component of the training file indicates the output class label. The flowchart of the proposed DNN-based model is shown in Figure 3.

Features were extracted from raw EEG signals using PSD as a feature extraction method. Power Spectral Density in band 8–30 Hz was calculated at a rate of 16 times per second, that is every 6.25 ms. The frequency resolution for obtaining PSD values was 2 Hz. The PSD method considered recoding data from eight centro-parietal channels C3, Cz, C4, CPI, CP2, P3, Pz, and P4. Therefore, the EEG data obtained are 96-dimension. All the data obtained together with the true class label of the mental task requested by the operator were given to the classifier as training data. The dataset contained a PSD sample per row. Thus, precomputed features contained 96 features as explained in Section 2.2 and the last column, i.e., the 97th component, specifies the corresponding mental task label requested by the operator.

### 3.2. Data Pre-Processing

The PSD values obtained during the feature extraction step are now pre-processed. After pre-processing they are provided as an input to the Deep Neural Network. The following steps describe the pre-processing.

Step 1: For each of the subjects, the training files were re-arranged according to the mental task performed.
Step 2: The average power of an EEG signal in the given frequency range was computed for each subject in the three training files. Since the records are arranged according to the task performed, averaging was done for a similar task. Thus, we obtained three averaged files, one for each subject.
Step 3: Then, the mean of averaged PSD values in a pair of two subjects was computed. Thus, we obtained three training files: average PSD for the first subject and second subject, average PSD for the second subject and third subject, and average PSD for the first subject and third subject. These steps are sorted task-wise, i.e., for subject 1, we have three tasks, for each task the trial is performed and activity is observed and recorded, and similarly for subject 2. However, we used the task-wise average. The task-wise averaging means that the right hand movement of subject 1 and right hand movement of subject 2 were considered and the averaged values were kept for further proceeding. Similarly, when considering left hand movement (LHM), the LHM of the two subjects under considerations were utilized.
Step 4: The leftover averaged file was used in the testing model. For example, when the model was trained with averaged PSD of subject 1 and subject 2, the PSD of subject 3 was utilized in the testing phase. Similarly, when the average PSD of subject 2 and subject 3 was considered for training purpose, the PSD of subject 1 was used for testing, etc.

**Figure 4:**
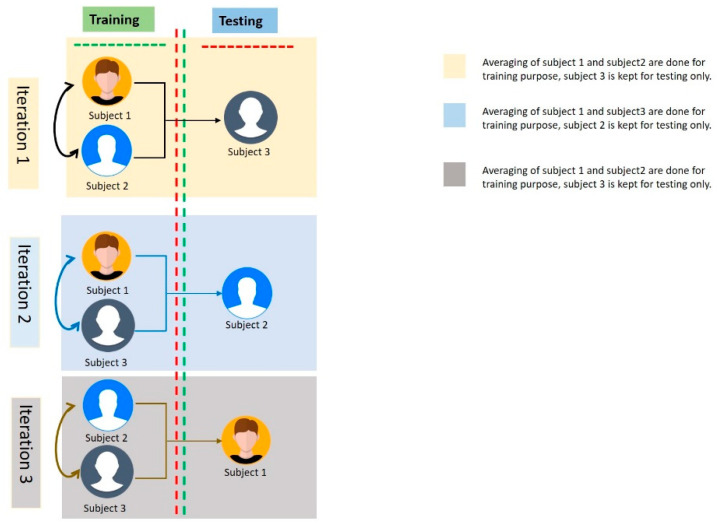
Illustration of how different subjects were considered for training and testing purposes. The figure also clearly conveys that the fold used for averaging the testing set was not used in the training phase.

It is noteworthy that the fold used for the testing set was not used in the training phase. To illustrate this diagrammatically, we have sketched the process in Figure 4.

EEG is a time-domain brainwave and is very unlikely to perform perfectly in a single trial. Therefore, it is suggested to take an average of PSD values. As the training file is arranged according to the mental task performed, the average corresponds to a similar mental task. The calculation of average PSD values across different subjects aims to find PSD values at different electrode locations that can map a PSD value of a new subject in a corresponding mental task. During Step 1, it was ensured that the averages are calculated for similar mental tasks. Because the classifier is subject-independent, all but one subject’s EEG data were merged in a file, and the PSD properties of the combined data were then used as input for the deep neural network to perform the imagined mental activity. The data of the subject that was not used in the training phase was used as test data. As a result, the classifier was put to the test with data from a subject that it has not been trained on. The dataset obtained in Step 4 must be restructured in such a way that the subject data that were not included in the training phase were used during the testing stage of the model for subject independence. As a result, three evaluation datasets were achieved. PCA is then applied to these datasets in order to reduce the dimensions before feeding them as input to the DNN.

### 3.3. Model Architecture

The Keras over TensorFlow framework was used for the implementation of the proposed DNN-based model. The model is a deep learning network that is trained in a supervised manner from scratch. The model description, hidden layers, and neurons exploited are shown in Table 2. The loss function based on cross-entropy is minimized with stochastic gradient descent. The use of an optimization algorithm in a classifier based on deep learning drastically improves results. The Adam optimization algorithm [31] can be employed for iteratively updating network weights during the training phase. The Adam optimization algorithm is used in the proposed model together with stochastic gradient descent. The rationale for using Adam optimizers were as follows: first, it assures that the parameter’s learning rate is well-maintained. This significantly boosts performance on issues with sparse gradients. Second, the learning rates for each parameter are updated depending on the mean of the current gradient’s magnitudes for the weight, allowing the system to perform well with live and non-stationary data such as EEG. As a result, for each network parameter, a learning rate is maintained and is distinctly updated as learning progresses. In contrast, stochastic gradient descent works on static and fixed learning rates for all weight updates.

Dropout layers help in the DNN by removing inputs to a layer probabilistically. The removed inputs may be input variables of feature vectors or previous layer activations. It creates a simulation of a huge number of networks having different network compositions. It results in the robustness of nodes to inputs in DNN. The rate of dropout layer indicates the probability value for assigning every input to the layer as zero. In our DNN model, the dropout rate was set to 0.5 and the training epochs and batch sizes were 64 and 10, respectively. Weight regularization was applied for the reduction of overfitting of a DNN with the training data, which in turn improved the performance of the model. Changes in the number of hidden layers and the number of neurons in the hidden layers were also used to experiment with different DNN topologies. The averaged data of all subjects are used as the training data for all observations. Two subjects at a time were used for training and the remaining one for testing, and then the training and testing subjects were changed. For example, as shown in Figure 3, first subject 1 and subject 2 were considered for training and subject 3 was used for testing. Then, for next iteration, we considered subject 2 and subject 3 for training, and subject 1 for testing, and so on. The top results were attained at five hidden layers, as shown in Table 2. There are various categories for weight regularization. They are L1 and L2 vector norms that need a hyperparameter to be configured. L1 regularization signifies the sum of the absolute weights and L2 regularization signifies the sum of the squared weights.

## 4. Experimental Results

First, we defined the performance metrics as given in previous studies [23,24,25,26,42,43,44,45].

Precision, the percentage of labels that were correctly predicted is represented by the model precision score. Another name for precision is the positive predictive value. False positives (Fp) and false negatives are traded off using precision together with the recall.

$$Precision=\frac{Tp}{Tp+Fp}$$

Recall—the model’s accuracy in predicting positives as distinguished from actual positives—is measured by the model recall score. This differs from the precision, which counts how many of the total number of positive predictions produced by the models are truly positive. Another name for recall is sensitivity or the true positive (Tp) rate. The model’s ability to recognize positive instances is demonstrated by a high recall score.

$$Recall=\frac{Tp}{Tp+Fn}$$

F1 Score—the model score as a function of the recall and accuracy is represented by the model F1 score. As an alternative to accuracy measurements, the F-score is a machine learning model performance statistic that equally weights the precision and recall when assessing how accurate the model is.

$$F1\;Score=\frac{2\times recision\times Recall}{Precision+Recall}$$

Accuracy—the model accuracy is mathematically defined as the ratio of Tp and Tn to all the positive and negative observations, representing one of the most widely used performance metrics for machine learning classification models. In other words, the accuracy indicates the number of times our machine learning model predicted a result accurately out of all the predictions it made.

$$A\mathrm{ccuracy}=\frac{Tp+Tn}{Tp+Tn+Fp+Fn}\times 100$$

The usefulness of each module of the proposed DNN-based model was established by conducting a study on different criteria for the inputs to the model. The results of different input criteria were further compared with the presented model to show that it performed better.

The usefulness of each pre-processing through averaging over training data in the proposed model was inspected here on the three datasets described in Section 2.4 with the corresponding accuracy values and F1 scores. The results are summarized in Table 3. The model settings “cross-subject” and “train averaged” represents models with cross-subject training settings without any averaging for data, and models with averaged data in the training stage, respectively. The necessity of performing averaging of the training subjects was first studied and then the importance of cross-subject averaging was emphasized. The comparison results in Table 3 indicate the importance of averaging testing and training data before providing input to the deep neural network. After analyzing the results of each test subject data, the proposed DNN-based model achieves a mean accuracy above 77%. The best result obtained is 85.7% with the data from subject 1 as the test data. It was also noted that the proposed model had varied accuracy scores as the test subjects were changed in experiments. The key cause is that EEG signals have high variability with diverse subjects and in some cases, there is a possibility that a particular subject was unable to accomplish the said tasks during the EEG signal recording.

Based on the values obtained as shown in the confusion matrices in Table 4, Table 5 and Table 6, we can calculate the true positive rate and the true negative rate using the formula defined and shown in Table 7.

## 5. Discussion

Table 8 shows an in-depth comparison of the proposed method with the most recent methods. For a reasonable evaluation, the most recent work that has an application code accessible on the Web was carefully chosen. The comparison was carried out with the EEGNet [10] based on the EEG feature extraction method. The CTCNN (Cropped Training CNN) method [46] is based on different convolutional networks with the suggestion of the crop training method. The EEG Image [47] method is based on spatial, temporal, and spectral features and deep learning, while AE-XGboost [48] and FBCSP [49] employ a traditional classification method in EEG analysis for the classification of mental tasks. Table 3 shows that the performance of the proposed model was clearly above the other approaches based on the accuracy and F1 score.

In addition to accuracy, FPR and FNR were also obtained for the proposed approach. The results showed that high accuracy for mental task classification has yet to be achieved high; however, with the state-of-the-art comparison, the proposed approach obtained slightly better results with an accuracy above 75% (0.7762).

**Figure 5:**
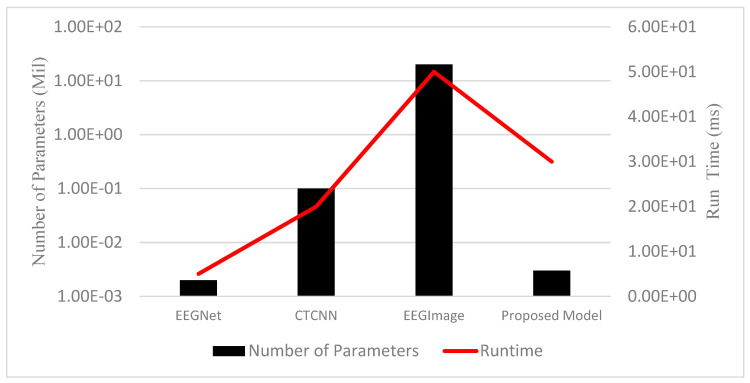
Comparison based on runtime and trainable parameters.

The proposed DNN-based model was also compared in terms of the requirement of total trainable parameters and the corresponding runtime for all models and the results are given in Table 9. It clearly shows that the proposed model had a satisfactory requirement of trainable parameters and had a low runtime requirement. The same results are also shown graphically in Figure 5.

**Figure 6:**
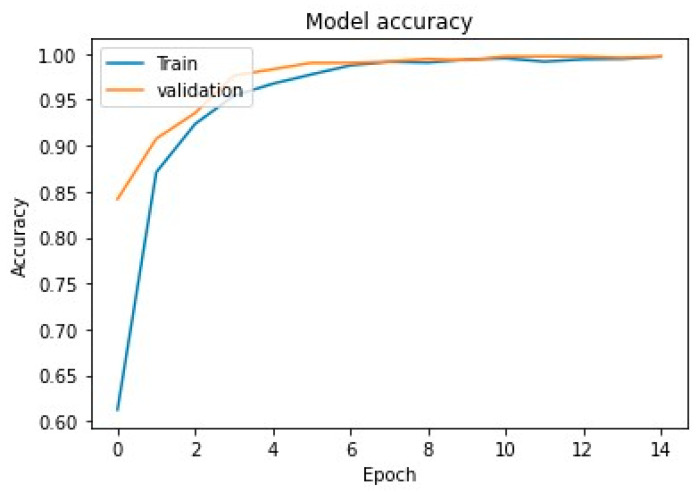
Model accuracy convergence of the model for training and validation.

**Figure 7:**
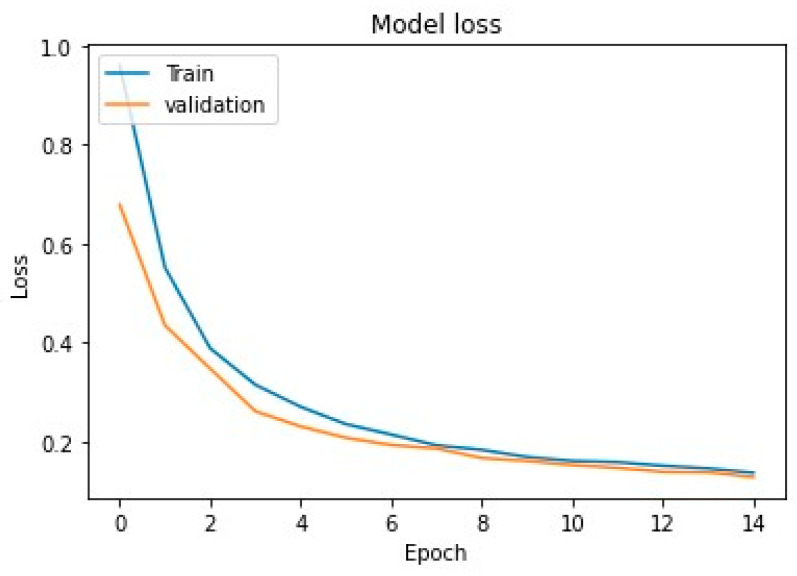
Model loss convergence of the model for training and validation.

In addition, we have also analyzed the convergence of the proposed model throughout the testing and training phases. Figure 6 depicts that, as the training epoch progresses, the accuracy of the training set first slowly increases and then finally stabilizes. Similarly, Figure 7 depicts that, as the training epoch progresses, the loss in the training set slowly decreases, demonstrating that the proposed model eventually converges in training with decent stability.

## 6. Conclusions

In the domain of BCI applications, the issue of subject-independent models is widely researched. The main challenge is to handle the high variability present in brain signals. The reason for the high variability is the involvement of the brain in other background tasks. During the imagination of a given mental task, the subject’s brain is also occupied with additional happenings. The observed brain signals are thus the output of the combination of these two tasks which is highly variable. The factors that affect the performance of the mental task can be attention, fatigue, or motivation. One of the major factors at the initial stage of the subject’s training is deviations in the policies the subjects make for performing the mental tasks.

This research focuses on mental task classification from EEG signals using a deep neural network. The proposed model is subject-independent, and therefore test subject data are not included in the training dataset for the model. The field of cross-subject EEG analysis is highly desired but has limited extant work. The proposed work suggests a DNN-based model for the analysis of EEG signals in a subject-independent way, that is, subject-independent mental task classification. We have averaged the PSD from the signals of all but one subject in the training phase. Once a deep learning model was trained, the PSD of the test subject was averaged with training data. This reduces the high variability of EEG signals across diverse subjects. The proposed subject-independent work was compared with the common benchmark dataset from BCI competitions. Different experimental setups and results indicate the significance of averaging training and testing data. Thus, the proposed model can be applied for the classification of mental tasks from the PSD values of EEG of any person whose data are not utilized during the training phase of the model. The only limitation could be the need to keep some training data for the testing phase as well. This work can be extended by building similar models with other deep learning models such as LSTM and bidirectional LSTM that are suitable for time series data (EEG).

We can further dive into the depths to explore other deep learning methods, and a few experiments can be performed to improve the accuracy. For example, factors that can influence the EEG signal data, possibly any noise or any disturbance caused by the cognitive aspects and hidden imbalanced state of an individual, would be of great interest.

## Figures and Tables

**Table 1:** Number of PSD samples for the three subjects.

	Number of Feature Vectors in the Training Dataset			Number of Feature Vectors in the Testing Dataset
	File 1	File 2	File 3	
**First Subject**	3488	3472	3568	3504
**Second Subject**	3472	3456	3472	3472
**Third Subject**	3424	3424	3440	3488

**Table 2:** The accuracy obtained for various DNN topologies using ‘Relu’ as activation function.

Number of Hidden Layers	Number of Neurons	Accuracy
3	12, 12, 12	0.780
3	12, 12, 24	0.806
3	24, 24, 12	0.791
3	24, 12, 24	0.778
5	12, 24, 12, 24, 12	0.807
6	12, 24, 12, 24, 12, 24	0.791

**Table 3:** Input criteria study for proposed DNN-based model.

Model Input Cross-Subject without Averaging	**Test**	**Evaluation Criterion**			
		**Accuracy**	**Precision**	**Recall**	**Fl**
	Subject 1	0.392027	0.430207	0.392028	0.401665
	Subject 2	0.346009	0.357656	0.34601	0.350267
	Subject 3	0.336554	0.336071	0.336547	0.342108
After Averaging	Subject 1	0.859479	0.865772	0.859348	0.857809
	Subject 2	0.67134	0.675895	0.671341	0.670794
	Subject 3	0.798715	0.830384	0.798713	0.800336

**Table 4:** Confusion matrix of subject 1.

Actual Values	**Predicted Values**			
	**Imaginary Task**	**Left Hand Movement**	**Right Hand Movement**	**Word Generation**
	Left hand Movement	757	71	57
	Right hand Movement	112	1000	184
	Word Generation	10	49	1183

**Table 5:** Confusion matrix of subject 2.

Actual Values	**Predicted Values**			
	**Imaginary Task**	**Left Hand Movement**	**Right Hand Movement**	**Word Generation**
	Left hand Movement	528	198	85
	Right hand Movement	113	970	264
	Word Generation	222	192	851

**Table 6:** Confusion matrix of subject 3.

Actual Values	**Predicted Values**			
	**Imaginary Task**	**Left hand Movement**	**Right hand Movement**	**Word Generation**
	Left hand Movement	714	5	4
	Right hand Movement	188	825	38
	Word Generation	201	322	1126

**Table 7:** True Positive Rate (TPR) and True Negative Rate (TNR) values for subjects 1, 2, and 3.

Subject	TPR = TP/(TP + FN)	TNR = TN/(TN + FP)
Subject 1	0.858895706	0.918809884
Subject 2	0.68624014	0.825365854
Subject 3	0.778556822	0.889278411

**Table 8:** Comparison of proposed model with recent models.

Research Work	Contribution	Averaging Training Data	Accuracy
EEGNet [10]	EEG signals from different BCI paradigms	No	0.513
CTCNN [37]	Cropped training strategy	No	0.4767
EEG Image [38]	Multi-channel EEG time series	No	0.327
AE XGboost [39]	Apprehending the inconsistency of inter-class EEG data with inter-class and inter-person EEG signals	No	0.3318
FBCSP [40]	Filter Bank Common Spatial Pattern (FBCSP)	No	0.3569
Proposed	Averaging EEG data for subjects in the training and testing phases	Yes	0.7762

**Table 9:** Comparison based on runtime and trainable parameters.

Model	Number of Parameters (mil)	Runtime (ms)
EEGNet	2.0 × 10^−03^	5.0 × 10^+00^
CTCNN	1.0 × 10^−01^	2.0 × 10^+01^
EEGImage	2.0 × 10^+01^	5.0 ×10^+01^
Proposed	3.0 × 10^−03^	3.0 × 10^+01^

## Data Availability

The data can be obtained via personal request to the first author.

## Abbreviations

**Abbreviation**	**Full Form**
BCI	Brain Computer Interface
BiLSTM	Bidirectional Long Short-Term Memory Network
CNN	Convolutional Neural Network
EEG	Electro Encephalography
BPNN	Back Propagation Neural Network
HMM	Hidden Markov Model
PSO	Particle Swarm Optimization
PSD	Power Spectral Density
SVM	Support Vector Machine
LDA	Linear Discriminant Analysis
CSP	Common Spatial Patterns
SNR	Signal-to-Noise Ratio
FBCSP	Filter Bank Common Spatial Pattern
MIT	Motor Imagery task
DNN	Deep Neural Network
SGD	Stochastic Gradient Descent
ADAM	Adaptive Moment Estimation
ReLU	Rectified Linear Unit
PCA	Principal Component Analysis
TP	True Positive
TN	True Negative
FP	False Positive
FN	False Negative
TPR	True Positive Rate
TNR	True Negative Rate
